# Estimation of Ground Reaction Forces and Moments During Gait Using Only Inertial Motion Capture

**DOI:** 10.3390/s17010075

**Published:** 2016-12-31

**Authors:** Angelos Karatsidis, Giovanni Bellusci, H. Martin Schepers, Mark de Zee, Michael S. Andersen, Peter H. Veltink

**Affiliations:** 1 Xsens Technologies B.V., Enschede 7521 PR, The Netherlands; giovanni.bellusci@xsens.com (G.B.); martin.schepers@xsens.com (H.M.S.); 2 Department of Health Science and Technology, Aalborg University, Aalborg 9220, Denmark; mdz@hst.aau.dk; 3 Department of Mechanical and Manufacturing Engineering, Aalborg University, Aalborg 9220, Denmark; msa@m-tech.aau.dk; 4 Institute for Biomedical Technology and Technical Medicine (MIRA), University of Twente, Enschede 7500 AE, The Netherlands; p.h.veltink@utwente.nl

**Keywords:** ground reaction force and moment, inertial motion capture, inverse dynamics, gait analysis

## Abstract

Ground reaction forces and moments (GRF&M) are important measures used as input in biomechanical analysis to estimate joint kinetics, which often are used to infer information for many musculoskeletal diseases. Their assessment is conventionally achieved using laboratory-based equipment that cannot be applied in daily life monitoring. In this study, we propose a method to predict GRF&M during walking, using exclusively kinematic information from fully-ambulatory inertial motion capture (IMC). From the equations of motion, we derive the total external forces and moments. Then, we solve the indeterminacy problem during double stance using a distribution algorithm based on a smooth transition assumption. The agreement between the IMC-predicted and reference GRF&M was categorized over normal walking speed as excellent for the vertical (*ρ* = 0.992, rRMSE = 5.3%), anterior (*ρ* = 0.965, rRMSE = 9.4%) and sagittal (*ρ* = 0.933, rRMSE = 12.4%) GRF&M components and as strong for the lateral (*ρ* = 0.862, rRMSE = 13.1%), frontal (*ρ* = 0.710, rRMSE = 29.6%), and transverse GRF&M (*ρ* = 0.826, rRMSE = 18.2%). Sensitivity analysis was performed on the effect of the cut-off frequency used in the filtering of the input kinematics, as well as the threshold velocities for the gait event detection algorithm. This study was the first to use only inertial motion capture to estimate 3D GRF&M during gait, providing comparable accuracy with optical motion capture prediction. This approach enables applications that require estimation of the kinetics during walking outside the gait laboratory.

## 1. Introduction

Assessment of ground reaction forces and moments (GRF&M) is an important stage in the biomechanical analysis procedure. Conventionally, these measures are recorded using force plate (FP) systems, which, despite their high accuracy, have several significant limitations [1]. Firstly, the fixed position of the plates on the ground together with the requirement to step with the whole foot on the plate for a successful measurement may cause subjects to alter their natural gait pattern. Moreover, due to their high cost, most laboratories are equipped with one or a couple of FPs, which makes tracking many successive steps during overground walking impossible. In addition, the measurements are bounded by the laboratory space and cannot be performed outside this area, for example during daily life activities.

Towards ambulatory assessment of kinetics, previous studies have suggested the use of either pressure insoles [2,3,4] or instrumented force shoes [5,6]. The main difference between these two systems is that the former measures only a pressure distribution in the shoe, whereas the latter measures directly three-dimensional forces applied beneath the shoe. Such devices have enabled ambulatory estimation of ankle kinetics [7] and knee kinetics [8], in combination with inertial measurement units (IMUs) and linked segment models. Although these methods are ambulatory and have estimated GRF&M with relative RMS errors of (1.1±0.1)% [9], they suffer from certain limitations. The low durability and repeatability of the pressure insoles result in a drop in the reliability of the results [10]. As for the instrumented force shoe, it has been suggested that optimization is needed to decrease the size and weight of its wearable instrumentation and make it practical for recording sessions of extended durations [11,12].

Recent advances in biomechanical analysis techniques are allowing the estimation of GRF&M using only kinematic data [13,14,15,16,17,18,19]. When applied to gait analysis, a common problem that needs to be addressed is the distribution of the total external force and moment during periods of double foot support. Several methods have been previously proposed. Two studies proposed approaches based on artificial neural networks to determine the distribution of forces and moments [14,15]. Recently, another approach used a musculoskeletal model-based technique in which a dynamic contact model is used to solve the indeterminacy problem, without using training data [16,17,18]. In another study, Ren et al. introduced a distribution function called the smooth transition assumption, which is based on the observation that the GRF&M on the trailing foot change smoothly towards zero during the double stance phase of gait [19]. The latter assumption was further validated and adjusted to decompose the right and left GRF&M measured from a single force plate [20]. That study pointed out a limitation of the original smooth transition assumption, in which the center of pressure remains constant during the double support due to the use of the same functions for both horizontal moments and vertical force.

To apply kinetics prediction methods to kinematic data, most of the existing research uses optical motion capture (OMC). However, the increased accuracy and reduced size, power and cost of IMUs have enabled the assessment of segment orientation [21] and later full-body motion capture in laboratory-free settings. This technique delivers good accuracy in estimating human body kinematics, such as joint angles [22], and has been previously validated versus optical motion capture estimates [23]. Only a few studies have attempted to assess kinetics from kinematics using such inertial motion capture (IMC) systems. In a recent study, a top-down inverse dynamics approach was applied to estimate GRF&M and L5/S1 joint moments during trunk bending [24]. Another study used IMUs to estimate the joint forces and moments during ski jumping [25]. The common limitation of those studies is that they examined only the total external loads applied on both feet and are, therefore, inapplicable to gait analysis.

Therefore, the aim of this study was to develop a computational method to predict GRF&M, using only IMC-derived kinematics during gait. The method was evaluated for three walking speeds, by comparing the predicted GRF&M with the results of FP measurements. In addition, we performed two sensitivity analyses to investigate the effect of cut-off frequency on the estimated GRF&M, as well as to validate the choice of threshold velocities used in the gait event detection algorithm.

## 2. Methods

### 2.1. Experimental Protocol

Eleven (11) healthy male volunteers (age: 30.97 ± 7.15 years; height: 1.81 ± 0.06 m; weight: 77.34 ± 9.22 kg; body mass index (BMI): 23.60 ± 2.41 kg/m${}^{2}$) participated in the measurements performed at the Human Performance Laboratory, at the Department of Health Science and Technology, Aalborg University, Aalborg, Denmark. The experiment was performed in accordance with the ethical guidelines of The North Denmark Region Committee on Health Research Ethics, and participants provided full written informed consent, prior to the experiment.

**Figure 1:**
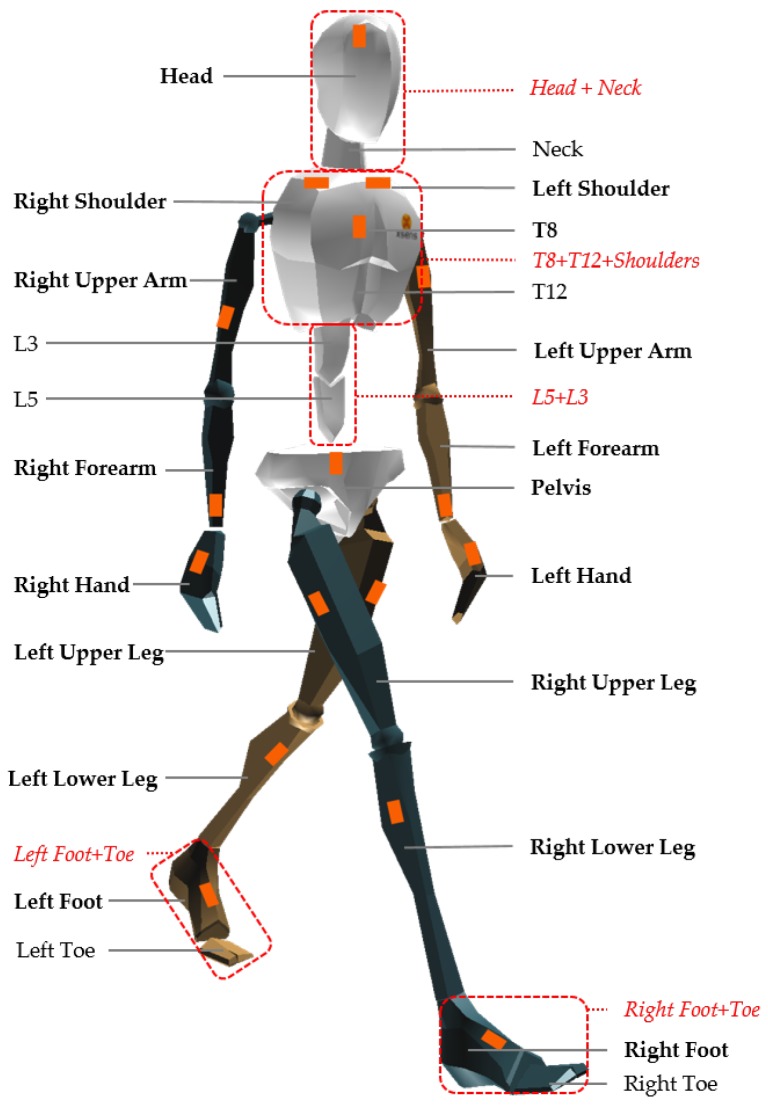
The definition of the 23 segments in the kinematic model of Xsens MVN. An inertial measurement unit is mounted on each of the 17 segments indicated with bold text. The 5 new segments (red italic font) are formed by the combination of MVN segments (within dashed-lined boxes) to match the segment definitions of De Leva [29].

**Figure 2:**
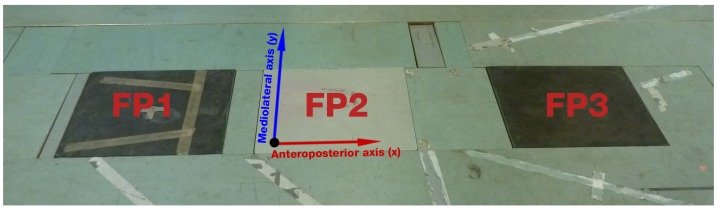
The three AMTI force plate system used, denoting the coordinate system of the laboratory. The vertical axis (*z*) points upwards, perpendicular to the anterior (*x*) and lateral (*y*) axes.

**Figure A1:**
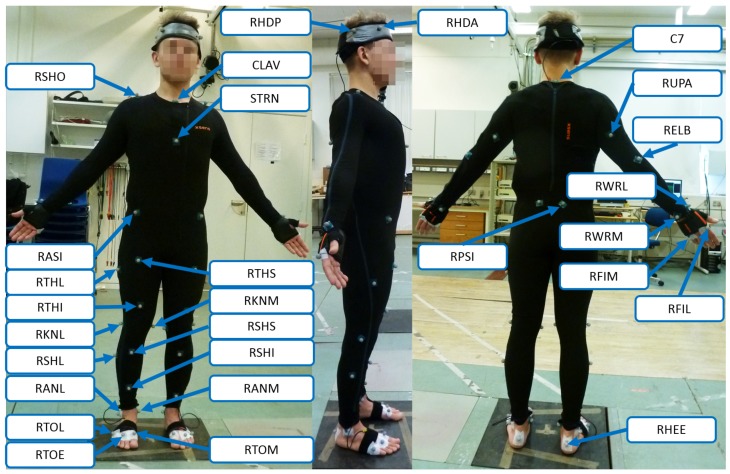
A subject in the wearable instrumentation, indicating the placement of the 53 retroreflective markers on the human body segments. All markers apart from clavicle, sternum and C7 are placed on the left side in a mirrored way.

The core system used in this study is an IMC system (Xsens MVN Link, Xsens Technologies BV, Enschede, The Netherlands [26]) powered by the matching software (Xsens MVN Studio version 4.2.4), delivering data at 240 Hz. The 17 IMU modules were mounted on a tight-fitting Lycra suit on the following segments: head, sternum, pelvis, upper legs, lower legs, feet, shoulders, upper arms, forearms and hands [22,27] (Figure 1). In addition, an OMC system, including eight infrared high-speed cameras (Oqus 300 series, Qualisys AB, Gothenburg, Sweden [28]), was used to capture 53 retro-reflective markers mounted on the body. The placement of the markers on the body is shown in Figure A1 and described in Table A1 in the Appendix section. Furthermore, three FPs (AMTI, Advanced Mechanical Technology, Inc., Watertown, MA, USA), embedded in the floor of the laboratory, recorded GRF&Ms (Figure 2). A combination of the OMC and FP systems (lab-based system) was used as a reference for comparison to the IMC-derived GRF&M predictions. To synchronize the IMC-based and lab-based systems, the Xsens sync station was used. The sampling frequency of the camera-based system was set to 240 Hz and that of FPs to 2400 Hz.

Before starting the recordings, the body dimensions of each subject were assessed and applied in the Xsens MVN software. Particularly, the heights of the ankle, knee, hip and top of head from the ground, the widths of the shoulder and pelvis, as well as the length of the foot were measured using a conventional tape with the subject in an upright posture [30]. These measurements were used to calibrate the IMC system using a steady upright posture, known as the neutral pose or n-pose [22]. The software classifies the quality of the calibration as “poor”, “fair”, “acceptable” or “good” based on the steadiness of the subject and the homogeneity of magnetic field around the IMUs at the time of the calibration. The calibration process was repeated before each set of walking speed trials and until the indication “good” was achieved in all cases, to maximize the quality of IMC.

Between the completion of the instrumentation setup and the start of the measurements, the subjects were given a five-minute acclimatisation period, to feel comfortable in the wearable equipment. Throughout the whole experiment, subjects remained barefoot, without wearing any type of footwear, apart from a thin strap wrapped around each foot to firmly mount the inertial measurement unit on these segments.

Gait at a self-selected normal (NW), fast (FW) and slow (SW) speed were measured. FW and SW speeds were instructed to the subjects as at least 20% higher or lower than their mean baseline NW speed, respectively. Particularly, the actual mean walking speeds performed were 1.28±0.14 m/s for NW, 1.58±0.09 m/s for FW (NW + 23%) and 0.86±0.11 m/s for SW (NW − 33%). To prevent the generation of additional external forces, the use of handrails or contact with any other external objects was not allowed. Before each task was recorded, subjects were given oral instructions and practiced the respective movement patterns. At least five successful trials per walking speed were obtained. A trial was considered successful when the right (left) foot hit one of the FPs completely, followed by a complete hit of the left (right) foot on the next FP. This definition ensures that FPs capture both right and left feet successfully within a stride.

### 2.2. Data Processing: IMC System

Xsens MVN estimates the orientation of segments by combining the orientations of individual IMUs with a biomechanical model of the human body. The orientation of each IMU is obtained by fusing accelerometer, gyroscope and magnetometer signals using an extended Kalman filter [31]. To relate the sensor orientations to segment orientations, a sensor-to-segment calibration procedure is performed. In this procedure, called n-pose, the subject is asked to stand in a known n-pose for a few seconds. The estimated transformation is applied and considered constant during a recording session.

We developed a program in MATLAB to assess the kinetic values from IMC-derived kinematics. By default, Xsens MVN Studio uses 17 IMU sensors to derive the kinematics of 23 segments as shown in Figure 1. Due to lack of literature reporting inertial parameters and relative center of mass positions for these exact segments, the original kinematic model has been adjusted to match the definitions of the 16-segment model reported by De Leva, 1996. To achieve this adaptation, 5 new rigid body segments were defined by merging specific given segments:

- Head-neck segment, formed by constraining the relative movement between head and neck segments. Kinematics were derived from the orientation of the IMU mounted on the head.
- Upper trunk segment, formed by constraining the relative movement between T8 and T12, T8 and right shoulder and T8 and left shoulder segments. Kinematics were derived from the orientation of the IMU mounted on the sternum.
- Middle trunk segment, formed by constraining the relative movement between L3 and L5 segments. Kinematics were derived from interpolation between the upper trunk and pelvis segment.
- Foot-toe, formed by constraining the relative movement between foot and toe segments. Kinematics were derived from the orientation of the IMU mounted on the foot.

The segment definitions of the pelvis, upper legs, lower legs, hands, forearms and upper arms remained unchanged (Figure 1; Table 1).

For the analysis, two coordinate systems were defined:

- the global coordinate system of the IMC system ($\psi_{g}$), in which the anterior axis points to the magnetic north, the vertical axis matches the direction of the gravitational acceleration and the lateral axis perpendicular to these axes, such that a right-handed coordinate frame is formed
- the walking coordinate system ($\psi_{w}$), which is defined by the same vertical, but has the anterior axes pointing in the walking direction, which means the difference between the two systems is only a rotation around the vertical; the walking direction was derived from known initial and final positions of the pelvis segment and assuming that the subjects walked approximately in a straight line throughout the trial

Knowing the kinematics and inertial properties of the segments of the biomechanical model, we estimated the total external force based on the Newton equations of motion:

(1) $$F_{ext}=\sum_{i=1}^{N}m_{i}\left(a_{i}-g\right)$$

where $F_{ext}$ denotes the total three-dimensional external force, *N* the total number of segments, $m_{i}$ the mass of each segment, $a_{i}$ the linear acceleration in the center of mass of each segment and g.

In a similar way, we calculated the total external moment from Euler’s equation:

(2) $$M_{ext}=\sum_{i=1}^{N}\left[J_{i}{\dot{\omega }}_{i}+\omega_{i}\times \left(J_{i}\omega_{i}\right)\right]-\sum_{i=1}^{N}\sum_{j=1}^{K_{i}}\left(r_{ij}\times F_{ij}\right)$$

where $M_{ext}$ denotes the total three-dimensional external moment, $K_{i}$ the number of end points in each segment (joints and external contact points), $\omega_{i}$ and ${\dot{\omega }}_{i}$ the angular velocities and angular accelerations of each segment, respectively. The inertia tensor around the center of mass of each segment is denoted by $J_{i}$, the position vectors between the center of mass and the end points denoted by $r_{ij}$ and the resultant force in the end points of each segment described by $F_{ij}$. All variables are expressed in the global coordinate system of the IMC system ($\psi_{g}$).

Segment linear accelerations exported from the Xsens MVN Studio software were expressed in the origin of each segment as described in detail in the manual of the IMC system [22]. To apply these variables in Equation (1), a translation to the segment’s center of mass was required, defined as:

(3) $$a_{i}=a_{i,o}+{\dot{\omega }}_{i}\times r_{oi}+\omega_{i}\times \left(\omega_{i}\times r_{oi}\right)$$

The position vectors between the center of mass and the origin of each segment $\left(r_{oi}\right)$ and the inertial parameters of the body segments $m_{i}$ and $J_{i}$ were calculated through scaled anthropometric data, based on adjustments to Zatsiorsky–Seluyanov’s inertial parameters reported by De Leva [29]. The total body mass of the subjects includes their actual body mass plus the mass of the wearable instrumentation. The added mass of the inertial motion capture system was in total 390 g (seventeen IMUs of 10 g each, one wireless communication pack of 150 g and one battery of 70 g). These additional masses were initially subtracted from the total measured mass before calculating the net mass of each segment and then added individually to each of the corresponding body segments. The resulting mass was input to the calculation of the moment of inertia from the radii of gyration. The effect of the wearable equipment in the radii of gyration was assumed to be negligible.

Segment angular velocities $\left(\omega_{i}\right)$, angular accelerations $\left({\dot{\omega }}_{i}\right)$ and linear accelerations of the origins $\left(a_{i,o}\right)$, provided by Xsens MVN software, were filtered using a second-order Butterworth zero-phase low-pass filter with a cut-off frequency of 6 Hz.

Our major assumption was that the GRF&Ms are the only significant external loads present. Thus, the total external force (moment) derived from Newton (Euler) equations of motion (Equations (1) and (2)) balances the sum of forces (moments) applied on both left and right lower limbs:

(4) $$F_{L}+F_{R}=F_{ext}$$

and

(5) $$M_{L}+M_{R}=M_{ext}$$

where $F_{L}$ ($M_{L}$) and $F_{R}$ ($M_{R}$) are the ground reaction forces (moments) applied on the left and right foot, respectively.

**Figure 3:**
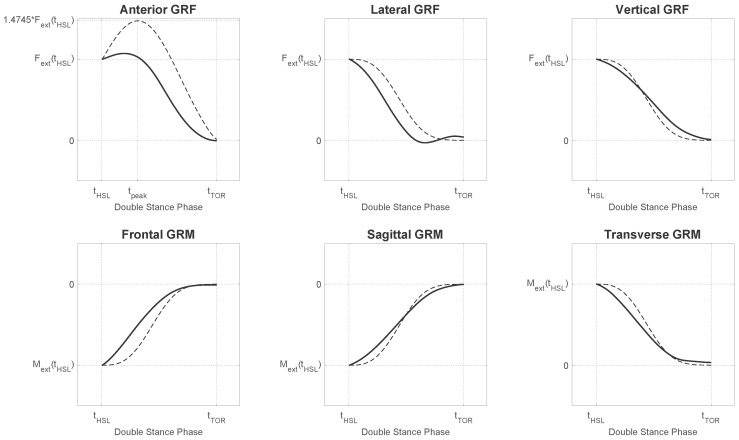
The curves of the smooth transition assumption function for the three GRF components ($f_{F,STA}$, three graphs on the top) and three GRM components ($f_{M,STA}$, three graphs on the bottom) used to distribute the total external force and moment among the two feet. Figure illustrates the curve of the GRF&Ms of the right foot between the events of left heel strike and right toe off (second double stance phase) expressed in the coordinate system defined by the walking direction. Continuous lines indicate the curves obtained from the average values across all subjects and trials of our dataset, whereas dashed lines indicate the curves proposed by Ren et al. [19].

During the single support phase, the result of the computation is the GRF&M applied on the foot, which is in contact with the ground. The resulting GRM is expressed about the external contact point on that foot, which is chosen as the projection of the ankle joint on the ground. However, during the phase of double support, the system of equations is indeterminate. To overcome this, we applied a distribution algorithm based on a smooth transition assumption function ($f_{STA}$), which was constructed from empirical data similarly to previous studies [19,20]. The curves of the measured GRF&M during the second double support phase were averaged for all steps. Subsequently, a cubic spline interpolation function was used to generate the $f_{STA}$. The generated function curves were compared to the ones proposed by Ren et al. [19] and shown in Figure 3. A direct comparison to the function of Villeger et al. [20] was not possible, because that study calculated the GRM about a fixed point on the plate and not with respect to the body.

The distribution function $f_{STA}$ was expressed in the coordinate system $\psi_{w}$. Since the input variables of the Newton–Euler equations were expressed in $\psi_{g}$, the same applied to the calculated vectors $F_{ext}$ and $M_{ext}$. Therefore, before applying the distribution function, we rotated the force and moment vectors from the coordinate system $\psi_{g}$ to $\psi_{w}$, resulting in two new vectors $F_{ext}^{w}$ and $M_{ext}^{w}$. The GRF&Ms applied on the left and right lower limbs are shown in Table 2, where $f_{F,STA}$ and $f_{M,STA}$ are the components of $f_{STA}$ for the GRF and GRM, respectively. Both functions depend on time (t) relative to the timing of gait events denoted by $t_{HSR}$, $t_{HSL}$ for heel-strike and $t_{TOR}$, $t_{TOL}$ for toe-off events for the right or left lower limb, respectively. The behavior of the components of $f_{F,STA}$ and $f_{M,STA}$ that was used in this implementation is illustrated in Figure 3.

**Figure 4:**
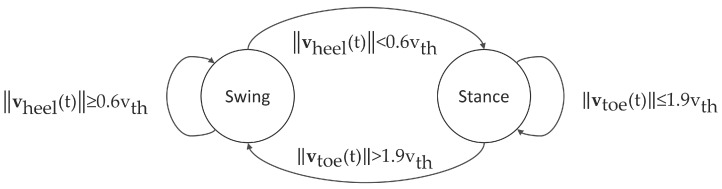
A state machine to detect the current state of the gait cycle, based on the previous state and a condition on the velocity of the heel or toe. The velocity $v_{th}$ is equal to the norm of the average velocity of the pelvis segment for each trial.

To distinguish between the phases of single and double stance, we used a gait event detection algorithm based on a threshold in the norm of the velocities of the heel ($\left||v_{heel}|\right|$) and the toe ($\left||v_{toe}|\right|$). The positions of the heel and toe points were provided by Xsens MVN Studio, and the velocity threshold ($v_{th}$) was set equal to the norm of the average velocity of the pelvis segment for each trial. The state of the gait cycle at time *t* is shown in Figure 4.

**Figure 5:**
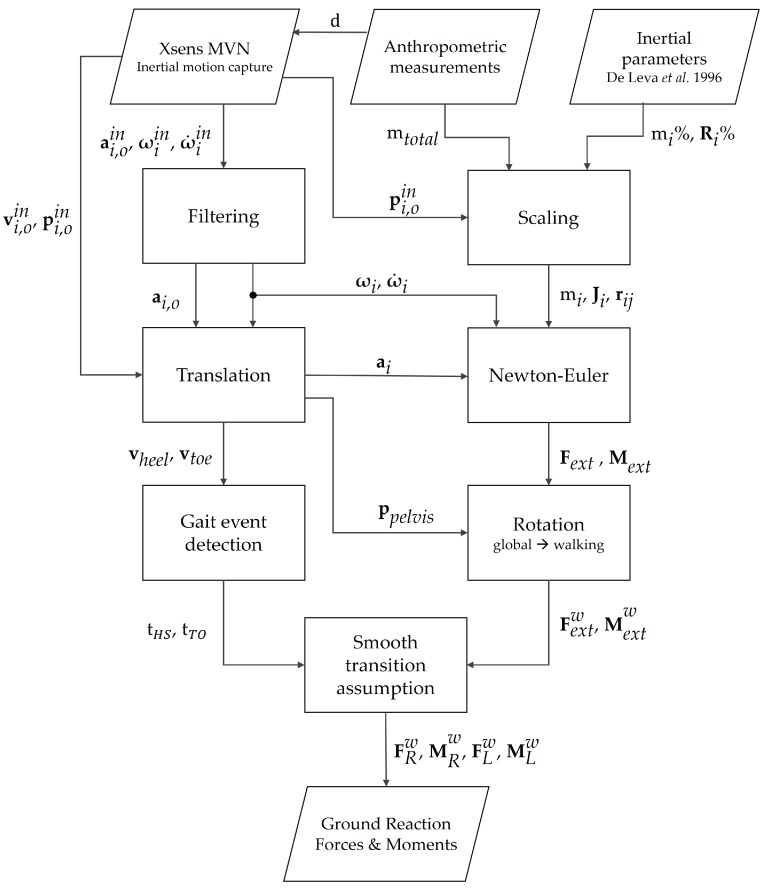
Block diagram of the algorithm used to estimate the GRF&M from anthropometry and inertial motion capture. p = position, v = velocity, a = acceleration, ***ω*** = angular velocity, $\dot{\omega }$ = angular acceleration, F = force, M = moment, *t* = time, *d* = anthropometric dimensions, *m* = mass, R = radius of gyration, J = inertia tensor. Superscript “in” indicates quantities derived directly from the IMC system, and *w* denotes quantities expressed in the coordinate system defined by the walking direction. Subscript *i* indicates a variable of the *i*-th segment. Additional subscript *o* denotes that a linear variable is expressed in the origin of the segment, whereas no additional subscript denotes that it is expressed in the center of mass of the segment. Subscript ext = external, *R* = right, *L* = left.

An overview of the algorithmic steps used in our study is shown in Figure 5.

### 2.3. Data Analysis: Reference Lab System

The Qualisys Track Manager 2.2 software package was used to process the three-dimensional positions of the markers and the GRF&Ms recorded using the FPs [28]. For each subject, a model for the automatic identification of markers was created, to assist the marker labeling across trials, and gaps were filled in the missing marker trajectories using a visual preview option. The marker trajectories were filtered using a second-order, zero-phase Butterworth low-pass filter with a cut-off frequency of 6 Hz, following the recommendations of the literature [1]. A biomechanical model composed of 16 segments, as described in Table 1, was constructed from the marker trajectories, with segment coordinate frames based on recommendations of the International Society of Biomechanics [32,33]. A custom script was developed in MATLAB R2015a (The MathWorks Inc.; Natick, MA, USA) to downsample the measured GRF&Ms by a factor of 10 to match the sampling rate of the OMC system. Similarly to Ren et al. [19], no filtering was applied to the processed GRF&Ms.

To compare the OMC data with the IMC data, all quantities expressed in the OMC lab coordinate frame ($\psi_{lab}$) were rotated to the coordinate frame based on the walking direction ($\psi_{w}$). Similar to the transformation applied to the IMC, $\psi_{w}$ is defined by a rotation around the vertical of $\psi_{lab}$, and the walking direction was derived from known initial and final positions of the pelvis segment.

In addition, the timing of the reference gait events (heel strike and toe-off) was identified and logged automatically, using a 5 N threshold on the vertical force measured by each FP. The steps that were only partially captured by the FPs were recognized and excluded based on the horizontal positions of the heel and toe markers during gait events. Due to the limited number of FPs, heel strike events used to denote the end of the gait cycle were not always available directly from the force data. In such cases, a velocity-based gait event detection, driven by the marker data, was used, similarly to Figure 4. The method performed with an error of 12 ± 10 ms in heel strike detection across all walking speeds.

The ground reaction force (GRF) was normalized to body weight and the ground reaction moment (GRM) to body weight times body height. In addition, for each step, the time was normalized to 100% of the gait cycle, defined by two consecutive heel contacts of the same foot. Finally, the six components of the measured and predicted GRF&Ms were compared, per walking speed and in total. The GRM was calculated about the projection of the ankle joint on the ground.

To evaluate the accuracy of our method, we used absolute (RMSE) and relative (rRMSE) root mean square errors, as defined by Ren et al. [19]. The agreement between the measured and predicted data normalized to the gait cycle was derived from Pearson’s correlation coefficients, which were categorized as weak (ρ≤0.35), moderate (0.35<ρ≤0.67), strong (0.67<ρ≤0.9) and excellent (ρ>0.9), according to previous studies [16,34].

Furthermore, we calculated the curve magnitude (M) and phase (P) percentage differences, based on the technique proposed by Sprague and Geers [35]. Out of 525 measured steps in total, 432 valid steps were included, and 93 steps were excluded due to incomplete stepping on the FPs.

In addition to the analysis over a whole gait cycle, we calculated the *ρ*, RMSE and rRMSE of the GRF&M throughout three sub-phases:

- DS1: first double stance phase of the ipsilateral foot, between a heel strike of the ipsilateral foot and a toe-off of the contralateral foot.
- DS2: second double stance phase of the ipsilateral foot, between a heel strike of the contralateral foot and a toe-off of the ipsilateral foot.
- SS: single stance phase of the ipsilateral foot, between a toe-off of the contralateral foot and heel strike of the contralateral foot.

Moreover, we analyzed the absolute and relative peak differences between the predicted and measured curves. The stance phase has been divided into three phases: early stance $\left(0\%<t/D_{stance}\leq 33\%\right)$, middle stance $\left(33\%<t/D_{stance}\leq 66\%\right)$ and late stance $\left(66\%<t/D_{stance}\leq 100\%\right)$, where $D_{stance}$ is the duration of the stance phase and *t* is the time initialized at the beginning of each stance phase. Within these phases, the following peaks have been sought for both predicted and measured values:

- In the early stance (ES) phase: the maximum values of lateral and vertical GRF and minimum value of anterior GRF.
- In the middle stance (MS) phase: the minimum value of the vertical GRF.
- In the late stance (LS) phase: the maximum values of the GRF components and transverse GRM and the minimum values of frontal and sagittal GRM components.

Finally, we compared the center of pressure (COP) and frictional torque estimates to the FP measurements. To derive these values for the right foot, we used the following equations:

(6) $$COP_{R,x}^{w}=-\frac{M_{R,y}^{w}}{F_{R,z}^{w}}$$

(7) $$COP_{R,y}^{w}=\frac{M_{R,x}^{w}}{F_{R,z}^{w}}$$

(8) $$COP_{R,z}^{w}=0$$

(9) $$T_{R,F}^{w}=M_{R,z}^{w}-COP_{R,x}^{w}F_{R,y}^{w}+COP_{R,y}^{w}F_{R,x}^{w}$$

where $COP_{R,x}^{w}$ and $COP_{R,y}^{w}$ are the anterior and lateral positions of the center of pressure on the ground with respect to the projection of the right ankle joint on the ground. $T_{R,F}^{w}$ is the frictional torque, $F_{R,x}^{w}$, $F_{R,y}^{w}$ and $F_{R,z}^{w}$ the anterior, lateral and vertical GRF, respectively, and $M_{R,x}^{w}$, $M_{R,y}^{w}$ and $M_{R,z}^{w}$ the frontal, sagittal and transverse GRM, respectively, calculated about the projection of the right ankle joint on the ground. The COP was calculated and analyzed per foot during stance phase, when $F_{R,z}^{w}$ was greater than 5 N. In the same way, the calculations for the left foot were performed. All variables are used in the equations individually for both right and left foot and are expressed in the coordinate system $\psi_{w}$.

In our implementation, the estimates of GRF&M depend highly on the performance of the gait event detection. Particularly, during the double support phase, the GRF&Ms applied on the ipsilateral foot are driven by the smooth transition assumption function. This is represented by a curve, which is based on the magnitude of each GRF&M component during the last single support frame and assumes zero magnitude on the last frame of double support. Thus, we evaluated the sensitivity of the heel strike and toe-off detection while using the original thresholds, compared to 10% higher and 10% lower than that.

We additionally performed a sensitivity analysis to investigate the effect of the selection of the cut-off frequency used in the second-order Butterworth low-pass filter. The change in the root mean square errors of each component of the GRF&M was used to indicate the impact of the selection in the final estimates.

## 3. Results

### 3.1. Accuracy Analysis

**Figure 6:**
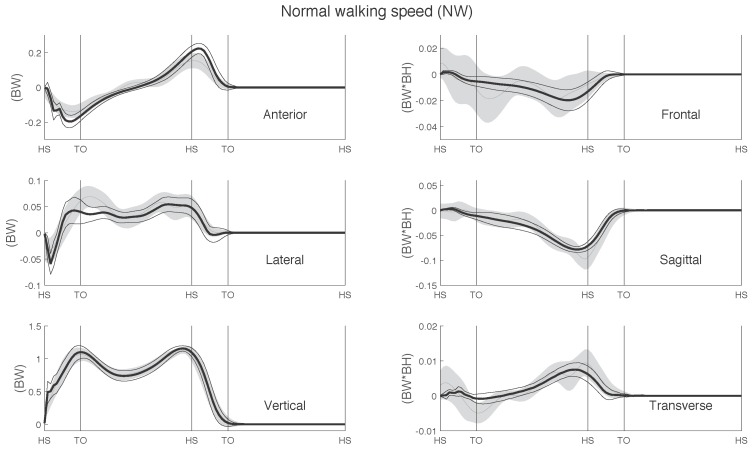
Ground reaction forces and moments (GRF&M) estimated using IMC (mean (thin grey line) ±1 SD around mean (shaded area)), compared with measured FP data (mean (thick black line) (±1 SD (thin black lines)) during normal walking. Curve magnitudes are normalized to body weight and body weight times body height for the GRF and GRM, respectively. Averaged over right and left steps of all 11 subjects.

**Figure 7:**
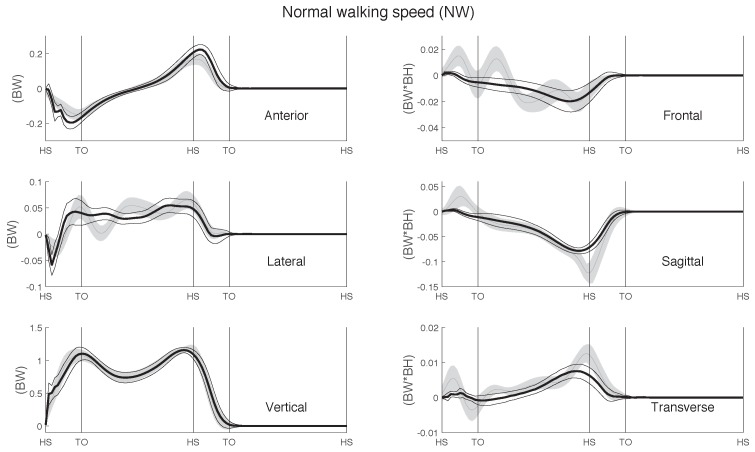
Ground reaction forces and moments (GRF&M) estimated using OMC (mean (thin grey line) ±1 SD around mean (shaded area)), compared with measured FP data (mean (thick black line) (±1 SD (thin black lines)) during normal walking. Curve magnitudes are normalized to body weight and body weight times body height for the GRF and GRM, respectively. Averaged over right and left steps of all 11 subjects.

The curves of the GRF&M throughout a whole gait cycle during normal walking, estimated via IMC and OMC, are depicted in Figure 6 and Figure 7, respectively.

Table 3 shows the results of the GRF analyzed throughout a whole gait cycle for both the IMC and OMC solutions. Overall, a similar performance by both systems can be observed for all metrics. Small differences were found in the anterior GRF where OMC provided higher accuracy (OMC: rRMSE = 7.4%, IMC: rRMSE = 9.4%). In contrast, similarly small differences were found in the lateral GRF, where IMC performed better. Table 4 shows the GRF estimates during the first and second double stance phase, as well as during the single support. Since the analysis is performed over a smaller period, higher absolute and relative errors are observed. Excellent correlations were found in all phases for both anterior and vertical GRF. The lateral GRF also presented excellent correlation during the second double support phase and strong correlations in the first double support phase. However, during the single support phase, the correlation is moderate and weak for IMC and OMC, respectively.

Similarly, the results for GRM are presented in Table 5 and Table 6 for both IMC and OMC solutions. For all walking speeds, the performance of IMC was comparable to OMC, except for the frontal plane moment where the rRMSE for IMC was between 29.6% and 30.6% across walking speeds, whereas for OMC, that was between 22.7% and 23.5%. In contrast, IMC provided higher correlations for the frontal plane GRM (*ρ* ranging from 0.709 to 0.710), compared to OMC (*ρ* ranging from 0.652 to 0.684) in all walking speeds. Excellent correlations were observed in the sagittal plane GRM for both normal walking (*ρ* = 0.933, rRMSE = 12.4%) and slow walking trials (*ρ* = 0.916, rRMSE = 13.3%). The correlation and RMSE in the transverse plane moment were similar in both solutions (IMC: *ρ* = 0.826, rRMSE = 18.2%, OMC: *ρ* = 0.825, rRMSE = 16.3%, for normal walking speed).

The center of pressure and frictional torque estimates are compared in Table 7. RMSE values were similar in both IMC and OMC solutions. The average RMSE of the anterior COP position ranges from 4.5 cm to 6.6 cm for IMC and from 4.4 cm to 6.5 cm for OMC. The average RMSE of the lateral COP position was ranging from 2.9 cm to 3.6 cm for IMC and from 2.4 cm to 2.7 cm for OMC. The estimates of the frictional torque were comparable for both solutions with rRMSE ranging from 23.5 to 27.6 for IMC and from 25.8 to 29.8 for OMC.

Finally, Table 8 presents the differences in the peak values of the estimated and measured GRF&M. As shown in the table, low relative errors have been extracted for the peaks of the vertical component for both solutions. In contrast, the differences in peak values were higher for the horizontal components.

### 3.2. Sensitivity Analysis

The sensitivity analysis on threshold velocities for the gait event detection algorithm is shown in Table 9. The gait events were detected using the inertial motion capture system with an error of 14.02±13.91 ms for heel strike and 16.09±15.68 ms for toe-off. A 10% increase or decrease in the threshold velocity would only result in a small additional error on the detection of both gait events (16.09±15.68 ms for 10% increase and 16.09±15.68 ms for a 10% decrease).

Table 10 shows the results of the sensitivity analysis performed to evaluate the selection of the cut-off frequency used in the low-pass filtering. Particularly, it indicates the change in the RMSE per component for six different cut-off frequencies. A low cut-off frequency selection leads to a decrease in the errors in the components with a small magnitude, such as the lateral GRF and the frontal and transverse GRM. In contrast, in the components with larger magnitudes, such as the vertical GRF and sagittal GRM, the accuracy increases with a higher cut-off frequency selection. To solve this inconsistency, the norms of the RMSE changes of the GRF and GRM 3D vectors were also compared. The norm of GRF RMSE is minimized for approximately 7 Hz and the norm of GRM RMSE for 6 Hz.

## 4. Discussion

In this study, we have developed a method to estimate 3D GRF&Ms during walking, using only kinematics from an IMC system. We evaluated the method for three different walking speeds performed by eleven healthy subjects. The accuracy of our estimates was assessed through comparison with force plate measurements, as well as with comparison to OMC-based estimates.

### 4.1. Comparison with Reported Optical-Based Estimation

In Table 11, we compare the performance of the IMC-based and OMC-based GRF&M estimation results during normal walking performed in the dataset of this study, with results previously reported in other optical-based studies using *ρ* and rRMSE.

In both the inertial and optical implementation of this study, we found higher correlation coefficients compared to the ones extracted from the 10-fold cross-validation method performed by Oh et al. [14] when they reimplemented the method of Ren et al. [19] for all components apart from the sagittal and transverse plane moments. Regarding rRMSE values, these followed a similar pattern, with the sagittal GRM providing slightly worse accuracy, whereas the transverse plane moment was better estimated in our method.

Compared to the dynamic contact model developed by Fluit et al. [16], we found similar correlation coefficients in all components, apart from the transverse plane, which was much higher in our case (IMC: *ρ* = 0.826; OMC: *ρ* = 0.825; Fluit et al.: *ρ* = 0.704). As for the rRMSE values, our technique performed similarly for four components (anterior, lateral, vertical, sagittal). The rRMSE in the transverse plane was lower in our findings (IMC: rRMSE = 18.2%; OMC: rRMSE = 16.8%; Fluit et al.: rRMSE = 40.60%). Fluit et al. explained that the cause of the inaccuracy in the transverse plane was the one-degree-of-freedom knee joint used. In contrast, the IMC method provided lower accuracy in the frontal plane GRM (IMC: rRMSE = 29.6%; OMC: rRMSE = 22.7%; Fluit et al.: rRMSE = 22.9%).

In the comparison with the results reported by the machine-learning-based method of Oh et al. [14], we noted similar correlations for the anterior (IMC: *ρ* = 0.965; OMC: *ρ* = 0.977; Oh et al. *ρ* = 0.985) and vertical (IMC: *ρ* = 0.992; OMC: *ρ* = 0.993; Oh et al. *ρ* = 0.991). The remaining components provided lower correlations and higher rRMSE in our findings.

The number of subjects included in our study was 11, higher than the previous optical-based studies, which included 3 [19], 5 [14] and 9 subjects [16]. This factor may have contributed to the larger standard deviations found in our RMSE values in anterior and vertical GRF.

In all four studies, regardless of the distribution technique used, the anterior and vertical GRF, as well as the sagittal GRM estimates performed better than the lateral GRF and frontal and transverse GRM. This behavior can be explained by the smaller magnitude of the lateral measures, which causes small accumulated errors in the input to have a relatively large impact on the final estimates. The majority of our results were in good agreement with the OMC-based literature.

This performance comparison demonstrates that IMC can be used in applications such as GRF&M prediction, performing similarly to OMC while exempting the restrictions of OMC mentioned previously in the Introduction.

### 4.2. Limitations and Sources of Error

In this study, we solved the indeterminacy problem during the double support phases of the gait cycle by utilizing a concept known as the smooth transition assumption. A function was generated from the average values of the force plate data during the second double support phase, similarly to Ren et al. [19]. Since these curves were obtained from the gait of 11 healthy subjects of this study, they may not be suitable for other groups, especially for populations with movement disorders. In addition, the differences found for various walking speeds indicate that a more sophisticated force distribution model is required. Therefore, methods based on either machine learning or dynamic contact models could be incorporated to improve the accuracy, repeatability and range of movements to which the method can be applied.

Filtering the input kinematics was necessary to obtain the best fit and reduce the errors in the GRF&M estimates. We translated the linear accelerations expressed in the origin to the center of mass of each segment. However, these accelerations were already translated from the sensor to the origin of the segment in Xsens MVN and include assumptions about the sensor location on the segment [36]. Moreover, angular accelerations were calculated through differentiation of angular velocities. This differentiation introduced high frequency signals, which require filtering before being used to translate accelerations. Nevertheless, it was demonstrated in the sensitivity analysis (Table 10) that any cut-off frequency between 5 Hz and 7 Hz would result in minor differences in the RMSEs.

The sensitivity analysis on the gait event detection, using the kinematics of the IMC system, proved that the algorithm is valid for the walking speeds included in this study. The algorithm resulted in an error of 14.05 ±13.91 ms, which for a sampling frequency of 240 Hz corresponds to 3.36 ± 3.33 samples. However, this algorithm may not be accurate for considerably slower or faster walking speeds or in cases of lower sampling frequencies. Gait misdetections could lead to considerable errors in the final estimates, so the method should be treated with caution.

In addition, mass ratios and radii of gyration of the body segments were estimated based on anthropometric tables found in the literature [29]. However, these parameters are averages and might not be suitable for all body types, for example for the elderly [37] or obese populations [38]. Therefore, inertia parameter approximations could have contributed to accumulative errors in the total external load estimation.

The IMC system uses a rigid-body linked-segment model in which the positions of the end points and joints were estimated through predefined measured lengths and IMU-derived segment orientations. The segment lengths were measured manually using a conventional measuring tape. Moreover, calibration limitations, such as a mismatch between the neutral pose practiced by the subject and the pose that the computational model is assuming, can cause errors. This limitation may explain the higher errors found in the frontal plane GRM in our solution, since it affects the estimates of the lever arms. Optical motion capture or photogrammetry could be used as an initial input to improve such offsets. Nevertheless, this implementation aimed to propose and investigate a completely laboratory-independent system.

Soft tissue artifacts are another common problem causing inaccuracies in both IMC and OMC kinematics [39,40]. The IMUs measure acceleration and angular velocity on the soft tissue, which moves relative to the bone [22]. This motion may have negative influence on our final estimates, especially in the case of fast walking. On the other hand, the fact that eight of the participants had a normal BMI (18.5 < BMI < 24.9), three were overweight (25 < BMI < 29.9) and no obese participants were included in the experiment probably limits the soft tissue effects in our study.

Finally, the IMC is susceptible to magnetic interferences. Particularly, it has been shown that the magnetic field varies considerably inside gait laboratories [41]. This factor may have influenced the sensor orientations used to derive the segment kinematics in Xsens MVN Studio software. Any input errors in the segment orientations could lead to accumulated errors in the estimated joint positions and, therefore, in the distance vectors between the center of mass and the joint of each segment. The latter are used in important stages of the proposed method, firstly in the translation of each segment’s linear kinematics from its origin to the center of mass (Equation (3)) and secondly in Euler’s equation of motion (Equation (2)). A magnetometer-free approach to inertial motion capture could be adopted to reduce these sources of error [42].

### 4.3. Future Work

In our experiment, we only included male subjects without any musculoskeletal or neuromuscular disorders. However, we did not evaluate the applicability to patients with motor-related clinical conditions. Several challenges could be encountered in the clinical application of the system. For example, in the case of knee osteoarthritis, the increased static knee misalignment of the patients might lead to difficulty achieving a proper neutral pose to calibrate the IMC system [43]. Moreover, obesity, which is quite common in patients with musculoskeletal problems, could impose practical barriers in the optical and inertial motion capture.

The smooth transition assumption we incorporated can only be applied to gait movements. On top of this, the distribution algorithm allows the real-time estimation only during the single support phase. During double support, the algorithm needs information over the duration of this phase to estimate the kinetics. A real-time solution to distribute the forces could be explored in the future.

In this study, we assume that the GRF&Ms are the only significant external forces applied to the human body. This assumption could be valid for activities such as walking; however, in a wider spectrum of daily life activities, secondary external loads are introduced. Such activities include walking using a cane or stair climbing using handrails. In such cases, direct measurement or modeling of the additional forces and moments would be required. Future work could examine the types and biomechanical importance of forces and moments generated in free-living environments, when performing daily life activities.

Finally, our proposed method is dependent on a full-body motion capture suit, which requires 17 IMUs. In future studies, minimizing the number of sensing modules [44] to make the system more practical for clinical and free-living applications could be investigated. Moreover, our system could be exploited in driving near real-time biofeedback, the popularity of which recently increased in gait training interventions [45].

## 5. Conclusions

In this paper, we have demonstrated that estimation of 3D GRF&Ms during walking using only kinematic information from inertial motion capture is achievable. Overall, strong and excellent correlations were found for all six estimated components compared to force plate measurements. The results were comparable to the ones reported by studies using OMC input.

The proposed system has high potential in monitoring critical biomechanical parameters in free-living conditions, outside the laboratory. Future work should validate and adapt the system to clinical and daily life applications.

## Figures and Tables

**Table 1:** Segments used in the 16-segment body biomechanical model, as described by De Leva [29], and the corresponding segments derived from the kinematic model of Xsens MVN Studio. The table includes the segment mass ratios (m), the longitudinal position of the center of mass in each segment ($CoM_{z}$), as well as the radii of gyration ratios ($r_{x}$, $r_{y}$, $r_{z}$).

De Leva 1996	Xsens MVN	m (%)	${\mathrm{CoM}}_{z}$ (%)	$r_{x}$ (%)	$r_{y}$ (%)	$r_{z}$ (%)
Definition	Equivalent					
Head	Head + Neck	6.94	50.02	30.3	31.5	26.1
Upper Trunk	T8 + T12 + Shoulders	15.96	50.66	50.5	32	46.5
Middle Trunk	L5 + L3	16.33	45.02	48.2	38.3	46.8
Pelvis	Pelvis	11.17	61.15	61.5	55.1	58.7
Upper Arm	Upper Arm	2.71	57.72	28.5	26.9	15.8
Forearm	Forearm	1.62	45.74	27.6	26.5	12.1
Hand	Hand	0.61	36.24	28.8	23.5	18.4
Upper Leg	Upper Leg	14.16	40.95	32.9	32.9	14.9
Lower Leg	Lower Leg	4.33	43.95	25.1	24.6	10.2
Foot	Foot + Toe	1.37	44.15	25.7	24.5	12.4

**Table 2:** The calculation of the left and right GRF&Ms for each phase of the gait cycle. During single support, the GRF&M of the limb in contact with the ground is equal to the result of the Newton–Euler calculation ($F_{ext}^{w}$,$M_{ext}^{w}$), whereas during the double support phase, this result is distributed among legs based on the gait-event-dependent three-dimensional smooth transition assumption functions for the forces ($f_{F,STA}\left(t\right)$) and moments ($f_{M,STA}\left(t\right)$).

Variable	First Double Stance	Right Single Stance	Second Double Stance	Left Single Stance
	$t_{\mathrm{HSR}}\leq t<t_{\mathrm{TOL}}$	$t_{\mathrm{TOL}}\leq t<t_{\mathrm{HSL}}$	$t_{\mathrm{HSL}}\leq t<t_{\mathrm{TOR}}$	$t_{\mathrm{TOR}}\leq t<t_{\mathrm{HSR}}$
$F_{L}^{w}\left(t\right)$	$F_{ext}^{w}\left(t_{HSR}\right)f_{F,STA}\left(t\right)$	0	$F_{ext}^{w}\left(t\right)-F_{R}^{w}\left(t\right)$	$F_{ext}^{w}\left(t\right)$
$M_{L}^{w}\left(t\right)$	$M_{ext}^{w}\left(t_{HSR}\right)f_{M,STA}\left(t\right)$	0	$M_{ext}^{w}\left(t\right)-M_{R}^{w}\left(t\right)$	$M_{ext}^{w}\left(t\right)$
$F_{R}^{w}\left(t\right)$	$F_{ext}^{w}\left(t\right)-F_{L}^{w}\left(t\right)$	$F_{ext}^{w}\left(t\right)$	$F_{ext}^{w}\left(t_{HSL}\right)f_{F,STA}\left(t\right)$	0
$M_{R}^{w}\left(t\right)$	$M_{ext}^{w}\left(t\right)-M_{L}^{w}\left(t\right)$	$M_{ext}^{w}\left(t\right)$	$M_{ext}^{w}\left(t_{HSL}\right)f_{M,STA}\left(t\right)$	0

**Table 3:** Comparison of ground reaction forces (GRF) estimated from inertial motion capture and optical motion capture versus force plate measurements. *ρ* = Pearson’s correlation coefficient, RMSE = root mean square error in N/BW, rRMSE = relative root mean square error (%), M = curve magnitude difference (%) and P = phase difference (%). Results are analyzed during normal (NW), slow (SW) and fast walking (FW) for the decomposed right and left GRF.

	Inertial Motion Capture			Optical Motion Capture		
	NW	SW	FW	NW	SW	FW
	*ρ*					
Anterior	0.965	0.955	0.950	0.977	0.974	0.977
Lateral	0.862	0.853	0.821	0.814	0.814	0.757
Vertical	0.992	0.990	0.986	0.993	0.991	0.987
	RMSE					
Anterior	0.034 (0.007)	0.036 (0.012)	0.047 (0.011)	0.028 (0.005)	0.029 (0.007)	0.034 (0.006)
Lateral	0.017 (0.003)	0.018 (0.005)	0.022 (0.004)	0.017 (0.003)	0.018 (0.005)	0.022 (0.004)
Vertical	0.063 (0.035)	0.075 (0.039)	0.090 (0.040)	0.058 (0.031)	0.067 (0.027)	0.081 (0.025)
	rRMSE					
Anterior	9.4 (2.5)	10.4 (3.2)	10.9 (3.1)	7.4 (1.5)	8.0 (1.9)	7.5 (1.3)
Lateral	13.1 (2.8)	13.8 (3.3)	14.6 (3.1)	14.2 (2.9)	14.2 (3.3)	15.5 (4.0)
Vertical	5.3 (3.1)	6.3 (3.3)	6.9 (3.0)	4.8 (2.7)	5.5 (2.2)	6.1 (1.8)
	M					
Anterior	−26.0 (10.5)	−28.8 (10.5)	−30.2 (9.9)	9.5 (3.2)	10.5 (3.5)	11.0 (3.1)
Lateral	23.1 (10.7)	24.6 (15.9)	28.8 (13.2)	14.1 (3.5)	14.7 (4.3)	16.1 (5.3)
Vertical	−1.0 (2.4)	−1.2 (1.9)	−1.5 (1.6)	3.1 (2.0)	3.6 (2.0)	4.2 (1.9)
	P					
Anterior	−22.0 (5.2)	−23.4 (5.9)	−22.5 (5.7)	7.2 (2.0)	7.7 (2.3)	7.1 (1.3)
Lateral	8.5 (9.6)	9.4 (11.0)	12.7 (12.6)	16.3 (3.7)	16.5 (4.7)	18.4 (5.7)
Vertical	0.3 (2.5)	0.3 (1.9)	0.6 (1.6)	2.8 (1.8)	3.2 (1.4)	3.7 (1.2)

**Table 4:** Comparison of ground reaction forces (GRF) estimated from inertial motion capture and optical motion capture versus force plate measurements. *ρ* = Pearson’s correlation coefficient, RMSE = root mean square error in N/BW, rRMSE = relative root mean square error (%). Results are analyzed for all walking speeds during the gait cycle phases of first double stance (DS1), second double stance (DS2) and single stance (SS) for the decomposed right and left GRF.

	Inertial Motion Capture			Optical Motion Capture		
	DS1	DS2	SS	DS1	DS2	SS
	*ρ*					
Anterior	0.918	0.976	0.975	0.921	0.983	0.993
Lateral	0.792	0.946	0.605	0.791	0.959	0.325
Vertical	0.946	0.995	0.980	0.936	0.997	0.984
	RMSE					
Anterior	0.058 (0.023)	0.066 (0.025)	0.033 (0.013)	0.056 (0.018)	0.055 (0.014)	0.018 (0.007)
Lateral	0.030 (0.012)	0.013 (0.007)	0.022 (0.007)	0.029 (0.011)	0.013 (0.009)	0.022 (0.007)
Vertical	0.143 (0.077)	0.118 (0.075)	0.042 (0.030)	0.149 (0.058)	0.092 (0.060)	0.033 (0.018)
	rRMSE					
Anterior	33.3 (10.8)	38.3 (16.5)	10.0 (3.6)	32.1 (8.6)	29.3 (9.6)	5.2 (1.6)
Lateral	30.9 (11.7)	25.5 (16.3)	35.4 (8.9)	29.7 (9.7)	22.9 (19.0)	34.6 (7.5)
Vertical	14.4 (6.7)	12.1 (8.6)	9.0 (5.2)	14.2 (4.7)	8.9 (9.1)	6.5 (2.4)

**Table 5:** Comparison of ground reaction moments (GRM) estimated from inertial motion capture and optical motion capture versus force plate measurements. *ρ* = Pearson’s correlation coefficient, RMSE = root mean square error in Nm/BW * BH, rRMSE = relative root mean square error (%), M = curve magnitude difference (%) and P = phase difference (%). Results are analyzed during normal (NW), slow (SW) and fast walking (FW) for the decomposed right and left GRM.

	Inertial Motion Capture			Optical Motion Capture		
	NW	SW	FW	NW	SW	FW
	*ρ*					
Frontal	0.710	0.707	0.709	0.684	0.675	0.652
Sagittal	0.933	0.916	0.841	0.942	0.932	0.880
Transverse	0.826	0.811	0.749	0.825	0.817	0.768
	RMSE					
Frontal	0.010 (0.004)	0.010 (0.004)	0.012 (0.004)	0.008 (0.001)	0.008 (0.002)	0.009 (0.002)
Sagittal	0.013 (0.004)	0.015 (0.006)	0.020 (0.005)	0.016 (0.006)	0.019 (0.008)	0.026 (0.006)
Transverse	0.003 (0.001)	0.003 (0.001)	0.004 (0.001)	0.002 (0.001)	0.003 (0.001)	0.004 (0.001)
	rRMSE					
Frontal	29.6 (9.3)	30.2 (9.3)	30.6 (8.0)	22.7 (4.1)	23.0 (4.6)	23.5 (4.9)
Sagittal	12.4 (3.4)	13.3 (3.8)	16.1 (3.2)	12.7 (3.5)	13.7 (3.8)	16.9 (2.7)
Transverse	18.2 (4.7)	18.8 (4.8)	21.6 (4.2)	16.8 (4.5)	17.6 (4.8)	21.0 (4.3)
	M					
Frontal	110.3 (146.3)	116.6 (135.7)	140.4 (116.1)	63.0 (92.0)	71.1 (94.9)	77.0 (84.8)
Sagittal	−0.7 (12.4)	5.3 (19.0)	22.5 (19.6)	36.1 (14.6)	41.2 (23.9)	63.9 (22.6)
Transverse	49.6 (28.3)	54.7 (33.7)	75.9 (33.8)	45.7 (27.5)	54.1 (37.1)	82.8 (40.9)
	P					
Frontal	19.7 (8.5)	21.5 (10.6)	21.0 (9.0)	23.8 (7.8)	24.7 (8.5)	25.2 (8.4)
Sagittal	13.1 (4.2)	13.8 (4.9)	16.9 (4.8)	10.1 (2.8)	11.2 (3.2)	13.9 (2.4)
Transverse	18.0 (5.8)	18.9 (6.2)	21.0 (6.7)	16.3 (3.9)	17.1 (4.7)	18.6 (5.0)

**Table 6:** Comparison of ground reaction moments (GRM) estimated from inertial motion capture and optical motion capture versus force plate measurements. *ρ* = Pearson’s correlation coefficient, RMSE = root mean square error in Nm/BW * BH, rRMSE = relative root mean square error (%). Results are analyzed for all walking speeds during the gait cycle phases of first double stance (DS1), second double stance (DS2) and single stance (SS) for the decomposed right and left GRM.

	Inertial Motion Capture			Optical Motion Capture		
	DS1	DS2	SS	DS1	DS2	SS
	*ρ*					
Frontal	0.556	0.803	0.431	0.515	0.951	0.472
Sagittal	−0.262	0.994	0.940	−0.137	0.997	0.943
Transverse	0.379	0.958	0.913	0.528	0.966	0.858
	RMSE					
Frontal	0.010 (0.004)	0.005 (0.004)	0.014 (0.006)	0.012 (0.005)	0.004 (0.003)	0.010 (0.003)
Sagittal	0.016 (0.007)	0.019 (0.017)	0.017 (0.006)	0.027 (0.011)	0.031 (0.023)	0.017 (0.007)
Transverse	0.004 (0.002)	0.003 (0.002)	0.003 (0.001)	0.005 (0.002)	0.004 (0.003)	0.003 (0.001)
	rRMSE					
Frontal	60.3 (17.7)	46.4 (28.4)	54.8 (17.8)	72.0 (30.7)	37.2 (32.2)	37.4 (7.4)
Sagittal	68.4 (16.2)	22.2 (14.9)	17.7 (5.2)	95.4 (25.7)	29.4 (22.1)	15.1 (4.1)
Transverse	56.2 (16.7)	31.3 (18.8)	23.3 (7.1)	61.8 (16.4)	35.2 (30.8)	19.8 (7.1)

**Table 7:** Comparison of center of pressure (COP) and frictional torque estimated from inertial motion capture and optical motion capture versus force plate measurements. *ρ* = Pearson’s correlation coefficient, RMSE = root mean square error in m for COP and Nm/BW * BH for frictional torque, rRMSE = relative root mean square error (%). Analysis performed over stance phase for normal (NW), slow (SW) and fast walking (FW) trials for the decomposed right and left quantities.

	Inertial Motion Capture			Optical Motion Capture		
	NW	SW	FW	NW	SW	FW
	*ρ*					
Anterior COP	0.803	0.777	0.526	0.884	0.818	0.702
Lateral COP	0.559	0.546	0.522	0.619	0.596	0.574
Frictional Torque	0.776	0.775	0.677	0.764	0.746	0.676
	RMSE					
Anterior COP	0.045 (0.013)	0.050 (0.018)	0.066 (0.016)	0.044 (0.010)	0.054 (0.016)	0.065 (0.012)
Lateral COP	0.029 (0.012)	0.031 (0.011)	0.036 (0.011)	0.024 (0.004)	0.025 (0.006)	0.027 (0.006)
Frictional Torque	0.004 (0.001)	0.004 (0.002)	0.005 (0.002)	0.005 (0.002)	0.005 (0.002)	0.006 (0.002)
	rRMSE					
Anterior COP	21.3 (5.3)	22.5 (6.9)	28.5 (5.7)	19.9 (3.4)	22.2 (5.0)	25.4 (4.0)
Lateral COP	32.4 (10.9)	34.5 (11.0)	37.2 (9.8)	28.3 (5.9)	28.6 (5.4)	29.2 (5.1)
Frictional Torque	23.5 (5.1)	23.9 (6.1)	27.6 (5.5)	25.8 (7.1)	26.5 (7.4)	29.8 (5.9)

**Table 8:** Differences in the peak values of the ground reaction forces and moments estimated using inertial motion capture and optical motion capture versus measured using force plates. Analysis performed for normal (NW), slow (SW) and fast walking (FW) trials for the decomposed right and left quantities. The subscripts ES, MS and LS indicate the phase of stance where each peak was found (early, middle and late stance phase, respectively).

	Inertial Motion Capture			Optical Motion Capture		
	NW	SW	FW	NW	SW	FW
	Absolute (N/BW)					
Anterior GRF $Min_{ES}$	0.051 (0.027)	0.055 (0.032)	0.086 (0.024)	0.049 (0.019)	0.051 (0.023)	0.071 (0.017)
Anterior GRF $Max_{LS}$	−0.072 (0.024)	−0.073 (0.032)	−0.100 (0.027)	−0.057 (0.016)	−0.058 (0.020)	−0.073 (0.020)
Lateral GRF $Max_{ES}$	0.026 (0.018)	0.024 (0.016)	0.023 (0.016)	0.000 (0.018)	0.001 (0.020)	0.002 (0.026)
Lateral GRF $Max_{LS}$	0.007 (0.014)	0.012 (0.020)	0.027 (0.024)	0.010 (0.015)	0.015 (0.022)	0.035 (0.024)
Vertical GRF $Max_{ES}$	−0.031 (0.016)	−0.031 (0.024)	−0.047 (0.023)	−0.018 (0.021)	−0.019 (0.025)	−0.036 (0.026)
Vertical GRF $Min_{MS}$	0.019 (0.011)	0.018 (0.012)	0.022 (0.012)	0.008 (0.008)	0.007 (0.007)	0.005 (0.009)
Vertical GRF $Max_{LS}$	−0.003 (0.035)	0.004 (0.046)	0.003 (0.055)	0.044 (0.047)	0.053 (0.064)	0.073 (0.078)
Frontal GRM $Min_{LS}$	−0.001 (0.013)	−0.005 (0.015)	−0.014 (0.016)	−0.002 (0.006)	−0.003 (0.008)	−0.007 (0.010)
Sagittal GRM $Min_{LS}$	−0.027 (0.020)	−0.033 (0.029)	−0.062 (0.025)	−0.053 (0.023)	−0.063 (0.035)	−0.098 (0.029)
Transverse GRM $Max_{LS}$	0.003 (0.003)	0.004 (0.004)	0.006 (0.005)	0.006 (0.003)	0.007 (0.006)	0.013 (0.005)
	Relative (%)					
Anterior GRF $Min_{ES}$	25.1 (11.8)	26.9 (15.8)	34.5 (11.9)	24.2 (7.7)	25.3 (8.0)	28.3 (7.1)
Anterior GRF $Max_{LS}$	−32.2 (12.6)	−32.5 (12.9)	−36.4 (9.4)	−25.0 (7.4)	−26.0 (7.6)	−26.5 (6.4)
Lateral GRF $Max_{ES}$	65.9 (70.4)	55.9 (59.4)	50.7 (65.7)	3.7 (38.4)	8.5 (43.0)	14.2 (54.2)
Lateral GRF $Max_{LS}$	15.5 (36.9)	32.5 (78.9)	79.6 (124.5)	19.8 (29.3)	42.5 (104.6)	111.2 (167.7)
Vertical GRF $Max_{ES}$	−2.8 (1.5)	−2.7 (2.0)	−3.8 (1.9)	−1.5 (2.0)	−1.6 (2.2)	−2.9 (2.0)
Vertical GRF $Min_{MS}$	2.7 (1.6)	2.8 (2.2)	4.2 (2.6)	1.1 (1.1)	1.0 (1.2)	1.0 (1.8)
Vertical GRF $Max_{LS}$	−0.2 (3.0)	0.4 (4.1)	0.4 (4.6)	3.8 (4.1)	4.7 (5.6)	6.2 (6.7)
Frontal GRM $Min_{LS}$	−10.9 (71.0)	−42.5 (134.5)	−115.8 (195.8)	−22.3 (52.6)	−36.8 (120.2)	−82.1 (194.1)
Sagittal GRM $Min_{LS}$	−34.3 (25.0)	−41.8 (36.6)	−76.2 (33.3)	−66.8 (28.4)	−79.5 (44.1)	−120.8 (39.3)
Transverse GRM $Max_{LS}$	46.2 (51.9)	49.9 (57.2)	72.5 (68.7)	80.6 (60.2)	102.3 (89.1)	169.0 (109.2)

**Table 9:** Sensitivity analysis on the threshold velocities used for the gait event detection.

	**Heel Strike Detection**		
Threshold velocity	$0.6v_{th}-10\%$	$0.6v_{th}$	$0.6v_{th}+10\%$
Mean error (ms)	18.87±15.44	14.02±13.91	15.37±14.35
	**Toe Off Detection**		
Threshold velocity	$1.9v_{th}-10\%$	$1.9v_{th}$	$1.9v_{th}+10\%$
Mean error (ms)	16.21±17.22	16.09±15.68	17.80±14.60

**Table 10:** Percentage change in the root mean square error (RMSE) of the three components and norms of the ground reaction force and moment, versus selected cut-off frequency for the second-order, zero-phase Butterworth low-pass filter. Negative values indicate improvement in the accuracy (decreased RMSE). The selected cut-off frequency (6 Hz) was used as a baseline for the comparison.

Frequency (Hz)	3	4	5	6	7	8	9
	RMSE change (%)						
Anterior	48.56	23.30	7.45	0.00	−1.48	0.57	4.41
Lateral	−13.78	−10.59	−5.39	0.00	7.72	18.07	29.89
Vertical	17.61	6.93	2.26	0.00	−1.02	−1.26	−0.96
Norm GRF	43.56	20.34	6.23	0.00	−0.52	2.45	7.23
Frontal	−14.94	−12.27	−6.51	0.00	7.78	15.76	24.79
Sagittal	61.80	29.54	8.58	0.00	−1.24	0.74	4.55
Transverse	−19.04	−11.35	−6.62	0.00	8.71	18.37	29.22
Norm GRM	40.75	17.22	3.76	0.00	1.90	6.09	11.88

**Table 11:** Pearson’s correlation coefficients (*ρ*) and relative root mean square errors (rRMSE) found in our IMC-based and OMC-based results and reported by previous studies based on optical prediction for normal walking [14,16,19]. The values marked with (*) are sourced from the 10-fold cross-validation performed by Oh et al. [14]. The number of subjects used in each study is denoted by *n*.

		Ground Reaction Force			Ground Reaction Moment		
	*n*	Anterior	Lateral	Vertical	Frontal	Sagittal	Transverse
		*ρ*					
This study (IMC)	11	0.965	0.862	0.992	0.710	0.933	0.826
This study (OMC)	11	0.977	0.814	0.993	0.684	0.942	0.825
Ren et al., 2008 *	3	0.878	0.704	0.913	0.677	0.978	0.829
Oh et al., 2013	5	0.985	0.918	0.991	0.841	0.987	0.868
Fluit et al., 2014	9	0.957	0.818	0.957	0.684	0.922	0.704
		rRMSE					
This study (IMC)	11	9.4 (2.5)	13.1 (2.8)	5.3 (3.1)	29.6 (9.3)	12.4 (3.4)	18.2 (4.7)
This study (OMC)	11	7.4 (1.5)	14.2 (2.9)	4.8 (2.7)	22.7 (4.1)	12.7(3.5)	16.8 (4.5)
Ren et al., 2008	3	10.9 (0.8)	20.0 (2.7)	5.6 (1.5)	32.5 (4.3)	12.2 (4.8)	26.2 (9.4)
Oh et al., 2013	5	7.3 (0.8)	10.9 (1.8)	5.8 (1.0)	22.8 (4.9)	9.9 (1.1)	25.5 (4.5)
Fluit et al., 2014	9	9.3 (2.0)	14.9 (3.4)	6.6 (1.1)	22.9 (5.9)	12.4 (3.5)	40.6 (11.3)

## Abbreviations

The following abbreviations are used in this manuscript:

BMI	body mass index
COP	center of pressure
FP	force plate
FW	fast walking
GRF	ground reaction force
GRM	ground reaction moment
GRF&M	ground reaction force and moment
IMC	inertial motion capture
IMU	inertial measurement unit
NW	self-selected normal walking
OMC	optical motion capture
rRMSE	relative root mean square error
RMSE	root mean square error
SW	slow walking

## Appendix A. Retroreflective Marker Protocol

**Table A1:** Marker protocol, indicating the marker labels and placement locations on the human body.

Label		Placement	Label			Placement
Right	Left		Right	Left	Center	
RHDA	LHDA	Head Anterior	RKNL	LKNL		Knee Lateral Epicondyle
RHDP	LHDP	Head Posterior	RKNM	LKNM		Knee Medial Epicondyle
RSHO	LSHO	Acromio-clavicular Joint	RSHS	LSHS		Shank Superior
RUPA	LUPA	Triceps Brachii	RSHI	LSHI		Shank Inferior
RELB	LELB	Elbow Lateral Epicondyle	RSHL	LSHL		Shank Lateral
RWRL	LWRL	Radial Styloid	RANL	LANL		Lateral malleolus
RWRM	LWRM	Ulnar Styloid	RANM	LANM		Medial malleolus
RFIL	LFIL	Second Metacarpal Head	RTOM	LTOM		First Metatarsal
RFIM	LFIM	Fifth Metacarpal Head	RTOE	LTOE		Third Metatarsal
RASI	LASI	Anterior Superior Iliac Spine	RTOL	LTOL		Fifth Metatarsal
RPSI	LPSI	Posterior Superior Iliac Spine	RHEE	LHEE		Calcaneus
RTHS	LTHS	Thigh Superior			C7	Seventh Cervical Vertebrae
RTHI	LTHI	Thigh Inferior			CLAV	Jugular Notch
RTHL	LTHL	Thigh Lateral			STRN	Xiphoid Process of the Sternum
